# LncRNA NEAT1 promotes nucleus pulposus cell matrix degradation through regulating Nrf2/ARE axis

**DOI:** 10.1186/s40001-021-00481-2

**Published:** 2021-01-21

**Authors:** Cheng Li, Xinjian Ma, Chenfei Ni, Jingyan Xu, Yinfei Xie, Junwei Kan, Xiaoli Wei

**Affiliations:** 1 Department of Rehabilitation Medicine, Affiliated Hospital of Jiangnan University, Wuxi, 214041 Jiangsu China; 2 Department of Traditional Chinese Medicine, Wuxi Guangrui and Tongjiang Community Health Service Center, Wuxi, 214000 Jiangsu China; 3 Department of Acupuncture, Affiliated Hospital of Jiangnan University (Formerly the Third People’s Hospital), No.585 Xingyuan North Road, Liangxi District, Wuxi, 214041 Jiangsu China

**Keywords:** LncRNA NEAT1, Intervertebral disc degeneration, Nrf2/ARE signaling pathway

## Abstract

**Background:**

This study aimed to assess the role and mechanism of lncRNA NEAT1 in intervertebral disc degeneration (IVD).

**Methods:**

LncRNA profile (GSE56081) between IVD and healthy control was downloaded from the Gene Expression Omnibus (GEO) database and analyzes differential lncRNA expression. Expression of lncRNA NEAT1 in IVD tissue and TNF-α/IL-1β-stimulated nucleus pulposus cells were further measured by RT-PCR. The lncRNA NEAT1 overexpression plasmids (pcDNA-NEAT1) were constructed and transfected into nucleus pulposus cells. Catabolic biomarkers (MMP-3 and MMP-13), anabolic biomarkers (Col II and Aggrecan) and Nrf2 expression were further measured. To further investigate the function of Nrf2, nucleus pulposus cells were pretreated with or without 25 μM tert-Butylhydroquinone (TBHQ), a Nrf2 activator, for 18 h and subsequently cotreated with pcDNA-NEAT1.

**Results:**

A total of 1432 lncRNAs were differentially expressed in GSE56081. Bioinformatic analysis found that these lncRNAs mainly enriched in Nrf2/ARE signaling pathway. LncRNA NEAT1 was highly expressed in IVD tissues than that of healthy control. Moreover, TNF-α/IL-1β induced a time- and dose-dependent increase in the mRNA expression of lncRNA NEAT1 in the nucleus pulposus cells. Overexpression of lncRNA NEAT1 abates promotes nucleus pulposus cells proliferation but induces matrix degradation. Meanwhile, nucleus and cytoplasm Nrf2 expression was significantly down-regulated by lncRNA NEAT1 upregulation. Nrf2 activator (TBHQ) could partially reverse the inhibitory effects of overexpression of lncRNA NEAT1 on matrix degradation.

**Conclusion:**

Collectively, our data unveiled the lncRNA NEAT1 promotes matrix degradation by regulating Nrf2/ARE signaling pathway, suggesting a potential therapeutic for IVD in the future.

## Introduction

Intervertebral disc degeneration (IVD) is mainly responsible for back pain and is a significant source of morbidity in our society [1, 2]. The risk factors for IVD comprise overload, obesity, genetic variants, and nucleus pulposus cell aberrant proliferation and finally resulted in increasing of inflammatory cytokines and matrix degradation [3, 4]. Treatment options for IVD include symptomatic management and/or surgery treatment [5, 6]. However, these treatments can only alleviate the symptoms, but cannot fundamentally improve the condition [7]. The pathogenesis of IVD is complicated and has not yet been fully elucidated. Thus, it is necessary to explore the molecular mechanism and pathogenesis of IVD and to search for therapeutic molecular targets.

Long intergenic noncoding RNA (LncRNA) is a growing family of transcripts of length > 200 nt that have a low protein-coding potential [8]. Accumulating evidences have shown that LncRNA play a vital role in cell proliferation, metabolism and apoptosis [9–11]. Meanwhile, lncRNAs are aberrantly expressed in IVD and thus speculated in the progress of IVD. For example, Chen et al. [12] reported that LINC01121 act as a sponge of miR-150-5p to induced IVD progression. Cui et al. [13] found that LncRNA MAGI2-AS3 is down-regulated in IVD, which was correlated with expression of FasL. LncRNA nuclear enriched abundant transcript 1 (NEAT1), a new lncRNA, has been demonstrated to serve an important role in cell apoptosis [14], non-small cell lung cancer invasion [15] and sepsis progression [16]. However, underlying mechanisms of NEAT1-regulating functions in IVD have yet to be elucidated.

The Nrf2/ARE signaling pathway have been shown to be involved in the regulation of cell proliferation, differentiation, migration and apoptosis [17, 18]. Nrf2 was modulated by PI3K/Akt signaling pathway which also played a crucial role in cell proliferation and apoptosis. Previous study revealed that Nrf2/ARE pathway was abnormally inactivated and thus speculated Nrf2/ARE signaling pathway may be used a treatment target for IVD degeneration [19]. However, whether lncRNA NEAT1 regulates nucleus pulposus cell proliferation through Nrf2/ARE signaling pathway was unknown.

In this study, we investigated differentially expressed lncRNAs between human nucleus pulposus (five degenerative as IVD and five normal specimens as control) from GSE56081. First, we proved that lncRNA NEAT1 was up-regulated in the degenerative nucleus pulposus (NP) tissues than normal specimens. Second, lncRNA NEAT1 overexpression induced cell growth and regulated extracellular matrix (ECM) expression in NP cell through regulation of Nrf/ARE pathway.

## Materials and methods

### Differential expression analysis

GSE56081 was downloaded from the NCBI GEO database (https://www.ncbi.nlm.nih.gov/geo/). In total, five degenerative samples and five normal specimens were used in this GEO dataset. Sequencing was performed on Arraystar Human LncRNA microarray V2.0 (Agilent_033010 Probe Name version) platform. The lncRNA expression profiles were first normalized by the *Z*-score and then subjected into analysis using this R package [20]. We identified differentially expressed lncRNAs with the limma package. Data visualization (heatmap and volcano plot) was performed using ggplot2 package [21]. KEGG pathway analysis was obtained from the R packages (R package, version 2.5.0).

### LncRNA NEAT1 subcellular localization

The subcellular localization of LncRNA NEAT1 in cells was identified with bioinformatics tools (http://lncatlas.crg.eu/) [22]. LncRNA NEAT1 subcellular localization in nucleus pulposus cells was detected by use of a FISH kit (Roche Applied Science, Germany) and nucleus was stained using DAPI (Solarbio, Beijing, China).

### Patients and samples

Normal intervertebral disc tissues (*n* = 20) were acquired from idiopathic scoliosis patients and degenerative intervertebral disc tissues (*n* = 60) were obtained from IVD patients that preparing for discectomy. Pfirrmann grades according to T2-weighted section images were used to assess the degree of IVD. According to Pfirrmann grading, grades I and II indicate normal discs, whereas grades III, IV, and V indicate degenerated discs [23].

### Cell culture

Human nucleus pulposus cells were isolated from normal intervertebral disc tissues of patients as described previously. In brief, intervertebral disc tissues were obtained and kept in the culture medium. Then, nucleus pulposus tissue was separated and cut into small pieces. Fragments were then digested in 0.2% type II collagenase at 37 °C for 4 h. Then, isolated cells were cultured in Dulbecco’s modified Eagle medium containing 20% fetal bovine serum (FBS, Gibco, Waltham, MA) in a 37 °C 5% CO_2_ incubator. The third generation of nucleus pulposus cells was used for next experiments. Different concentrations of tumor necrosis factor-alpha (TNF-α, 0, 10, 25 and 50 ng/ml) and interleukin-1β (IL-1β, 0, 5, 10 and 20 ng/ml) were used to induce degeneration of nucleus pulposus cells. Nucleus pulposus cells were grown to confluence and stimulated with TNF-α (50 ng/ml) or IL-1β (20 ng/ml) for 0, 24, 48 and 72 h.

### Cell transfection

The pcDNA-NEAT1 plasmid and pcDNA empty vector were constructed and purchased from Shanghai GeneChem Co., Ltd. Nucleus pulposus cells (1 × 10^5^) were seeded in a 6-well plate and then transfected with pcDNA-NEAT1 and pcDNA at a final concentration of 5 mg/l using Lipofectamine 2000 reagent (Invitrogen; Thermo Fisher Scientific, Inc.). After 24 h of transfection, the transfection efficiency was verified by qRT-PCR and the subsequent experimental procedure was performed. To further explore the molecular mechanism of lncRNA NEAT1 actions on nucleus pulposus cell proliferation, we activated the Nrf2 expression by tert-Butylhydroquinone (TBHQ) (Nrf2 activator, catalogue number: HY-100489, MCE, Shanghai, China) at final concentration of 25 μM for 18 h in vivo.

### Cell proliferation

The effect of lncRNA NEAT1 on nucleus pulposus cell proliferation was measured by CCK-8 method. In brief, 2000 nucleus pulposus cells from each group were seeded in a 96-well plate. After 0, 24, 48, 72, and 96 h of incubation, the viability of nucleus pulposus cells was analyzed by adding the CCK-8 solution (Hanheng Biotechnology Corp., Shanghai, China) in 10% volume. The absorbance of each well was determined by a microplate reader (Bio-Rad, Hercules, CA, USA) at 450 nm.

### PCR

Total RNA from nucleus pulposus cells was extracted using Trizol reagent (Vazyme, Nanjing, China). Total RNA template (500 ng) was reverse-transcribed into cDNA using a PrimeScript RT Master Mix Kit (RR036, Takara, Kyoto, Japan). Then, quantitative real-time RT-PCR (qRT-PCR) was performed on Roche LightCycler 96 System (CT, USA) using SYBR Premix EX TaqTM (RR420A, TakaRa, Kyoto, Japan). Data analysis was done using the 2^−△△*CT*^ method for relative quantification, and all samples were normalized to GAPDH. Primer sequences are provided in Table 1.

**Table 1:** Sequences of primers used for RT-PCR

Gene	Forward primers (5′-3′)	Reverse primers (5′-3′)
GAPDH	GGAGCGAGATCCCTCCAAAAT	GGCTGTTGTCATACTTCTCATGG
Col II	TGCTGCCTTTTCTGTTCCTT	AAGGTGCTGGGTAGGGAAGT
Aggrecan	ACATCTCAGCAGCATCATCACC	CATCACCACGCAGTCCTCAC
MMP-3	ATGCCCACTTTGATGATGATGAAC	CCACGCCTGAAGGAAGAGATG
MMP-13	CGGTTCCGCCTGTCTCAAG	CGCCAAAAGTGCCTGTCTT
lncRNA NEAT1	TGGCTAGCTCAGGGCTTCAG	TCTCCTTGCCAAGCTTCCTTC

### Western blot

Protein was extracted from nucleus pulposus cells with RIPA Buffer (Solarbio, Beijing, China). Nuclear proteins from nucleus pulposus cells were extracted using a Nucleoprotein Extraction Kit (Sangon Biotech Co., Ltd., Shanghai, China). Protein samples were boiled in SDS-PAGE loading buffer and subjected to SDS-PAGE. The proteins were then transferred on to a PVDF membrane followed by blocking with 2% non-fat milk powder in TBST. Subsequently, PVDF membranes were incubated with primary antibodies anti-MMP-13 (1:1000, ab39012, Abcam, USA), anti-MMP-3 (1:200 ab53015, Abcam, USA), Aggrecan (1:2000, ab3778, Abcam, USA), Col II (1:1000, ab34712, Abcam, USA), Nrf2 (1:5000, ab31163, Abcam, USA), Lamin (1:1000, ab16048, Abcam, USA) and GAPDH (1:1000, ab181602, Abcam, USA) overnight at 4 °C. The membranes were washed three times in TBST and incubated with horseradish peroxidase (HRP)-conjugated secondary antibodies (1:5000; cat. ab6721, Abcam, USA). The membranes were incubated with ECL substrate for 1 min and the Geliance 600 Imaging System (Perkin-Elmer) to quantify the exposed films. The relative level of total protein was normalized to GAPDH and the relative level of nuclear protein was normalized to Lamin.

### Statistical analysis

Three independent repetitions were performed for all experiments. Data were expressed as mean ± SD. One-way analysis of variance (ANOVA) followed by a Dunnett’s *t* test was performed for three and more groups. A *p* value less than 0.05 was considered to indicate statistical significance. Statistical analyses were performed by SPSS 20.0 (SPSS, Inc., Chicago, IL, USA).

## Results

### Bioinformatic analysis of GSE56081

**Fig. 1:**
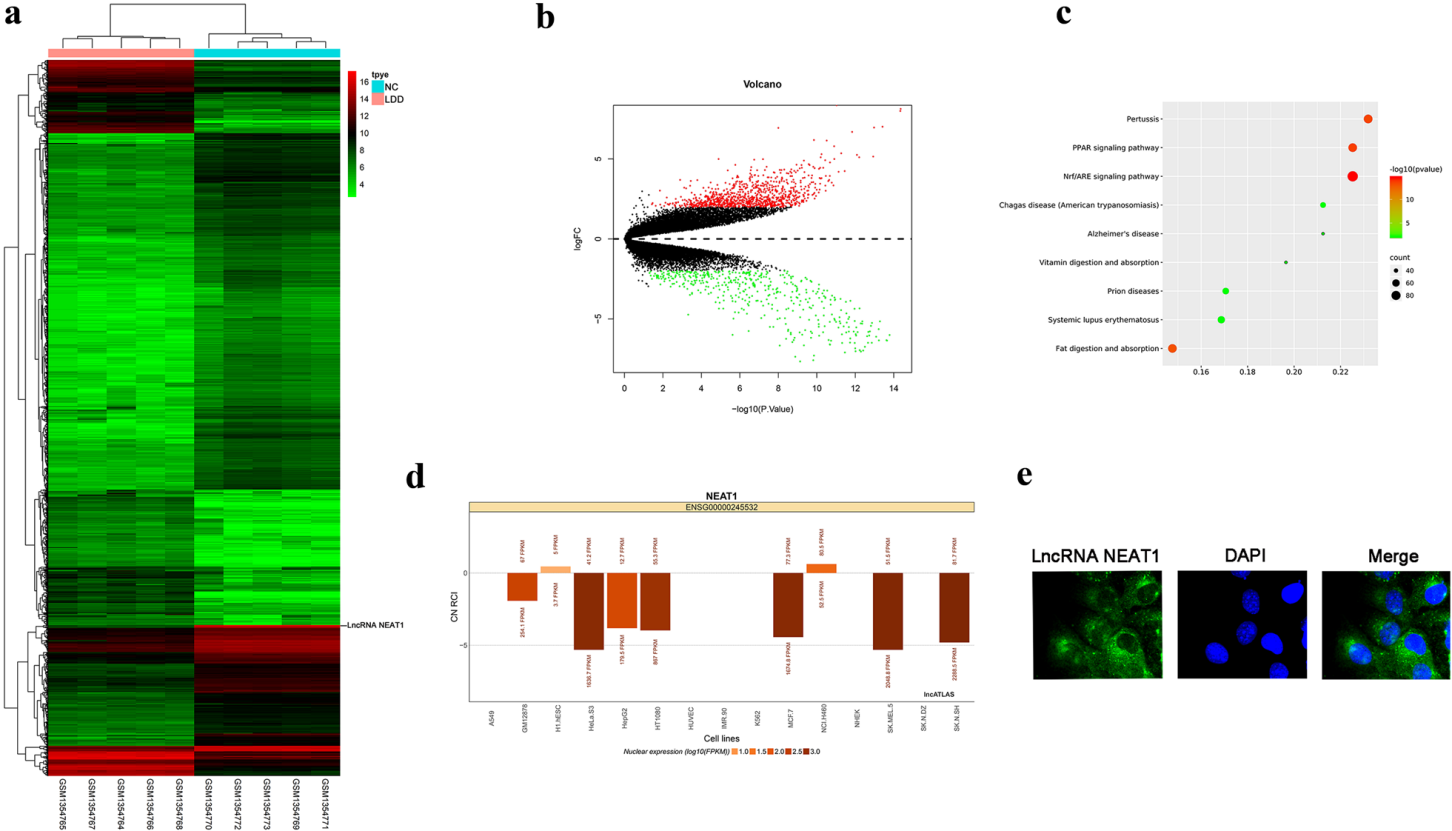
Bioinformatic analysis of GSE56081. **a** Heatmap of differentially expressed lncRNAs associated with IVD, red = high expression, green = low expression. **b** Volcano plot of differentially expressed lncRNAs associated with IVD, green dot represents down-regulated lncRNAs and red dot represents up-regulated lncRNAs in IVD tissue; black dot represents normally expressed lncRNAs. **c** KEGG pathway analysis for differentially expressed lncRNAs. **d** The abscissa represents gene ratio, and the ordinate represents enriched terms, the node color showed degree of enrichment ranging from low (green) to high (red), while the node size represented the frequency of the proteins in each enriched pathway group. **e** Subcellular localization plots of lncRNA NEAT1 displayed by lncATLAS. **f** Representative image of RNA FISH to confirm lncRNA NEAT1 location in nucleus pulposus cells (bars, 40 μm)

GSE56081, which including five degenerative samples and five normal samples, was used to identify the differentially expressed lncRNA. A total of 1432 lncRNAs were differentially expressed, among which 965 were up-regulated and 467 lncRNA were down-regulated (Fig. 1a, b). We selected lncRNA NEAT1 for further research mainly based on its expression was most statistically significantly. Top canonical pathways included Nrf/ARE signaling pathway, pertussis, PPAR signaling pathway, change disease, vitamin digestion and absorption, prion disease, systemic lupus erythematosus and fat digestion and absorption (Fig. 1c). LncRNA NEAT1 was mainly located in the nucleus, which were predicted by lncATLAS website (Fig. 1d) and further verified by FISH (Fig. 1e). Therefore, lncRNA NEAT1 can play a regulatory role as downstream gene expression.

### LncRNA NEAT1 has high expression in IVD tissue and cell models

**Fig. 2:**
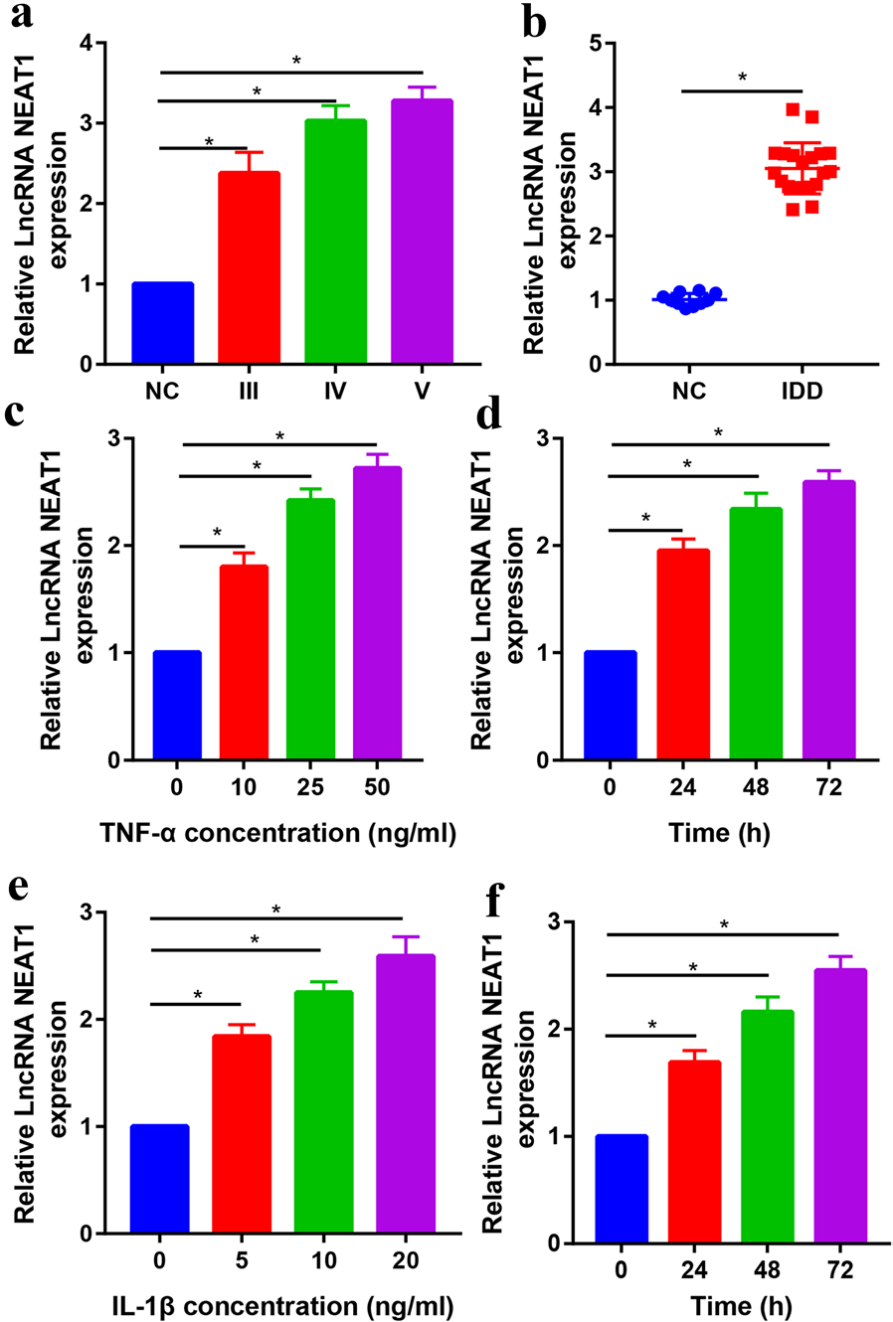
lncRNA NEAT1 was up-regulated in IVD tissue and NF-α/IL-1β-stimulated nucleus pulposus cells. **a** The lncRNA NEAT1 expression was increased upon elevated Pfirrmann grades. **b** The expression level of lncRNA NEAT1 was significantly increased in IVD tissues. **c** Expression of lncRNA NEAT1 in nucleus pulposus cells treated with different doses of TNF-α (0, 10, 25 and 50 ng/ml). **d** Expression of lncRNA NEAT1 in nucleus pulposus cells treated with TNF-α (50 ng/ml) at different time (0, 24, 48 and 72 h). **e** Expression of lncRNA NEAT1 in nucleus pulposus cells treated with different doses of IL-β (0, 5, 10 and 20 ng/ml). **f** Expression of lncRNA NEAT1 in nucleus pulposus cells treated with IL-β (20 ng/ml) at different time (0, 24, 48 and 72 h). *p < 0.05

To detect the gene expression of lncRNA NEAT1, we used real-time PCR to detect lncRNA NEAT1 mRNA expression levels in IVD tissue and TNF-α/IL-1β-stimulated nucleus pulposus cells at different time points. LncRNA NEAT1 expression level increased with the severity of the IVD increased (Pfirrmann grades: II–IV, Fig. 2a). Moreover, the expression of lncRNA NEAT1 was also significantly increased in IVD tissue when compared with normal tissues (Fig. 2b).

TNF-α and IL-1β were two common pro-inflammatory cytokines that used to establish the IVD cell model. With the increase in TNF-α or IL-1β concentration and duration, the lncRNA NEAT1 expression gradually increased with statistical significance (Fig. 2c–f).

### LncRNA NEAT1 induces the matrix degradation of nucleus pulposus cells

**Fig. 3:**
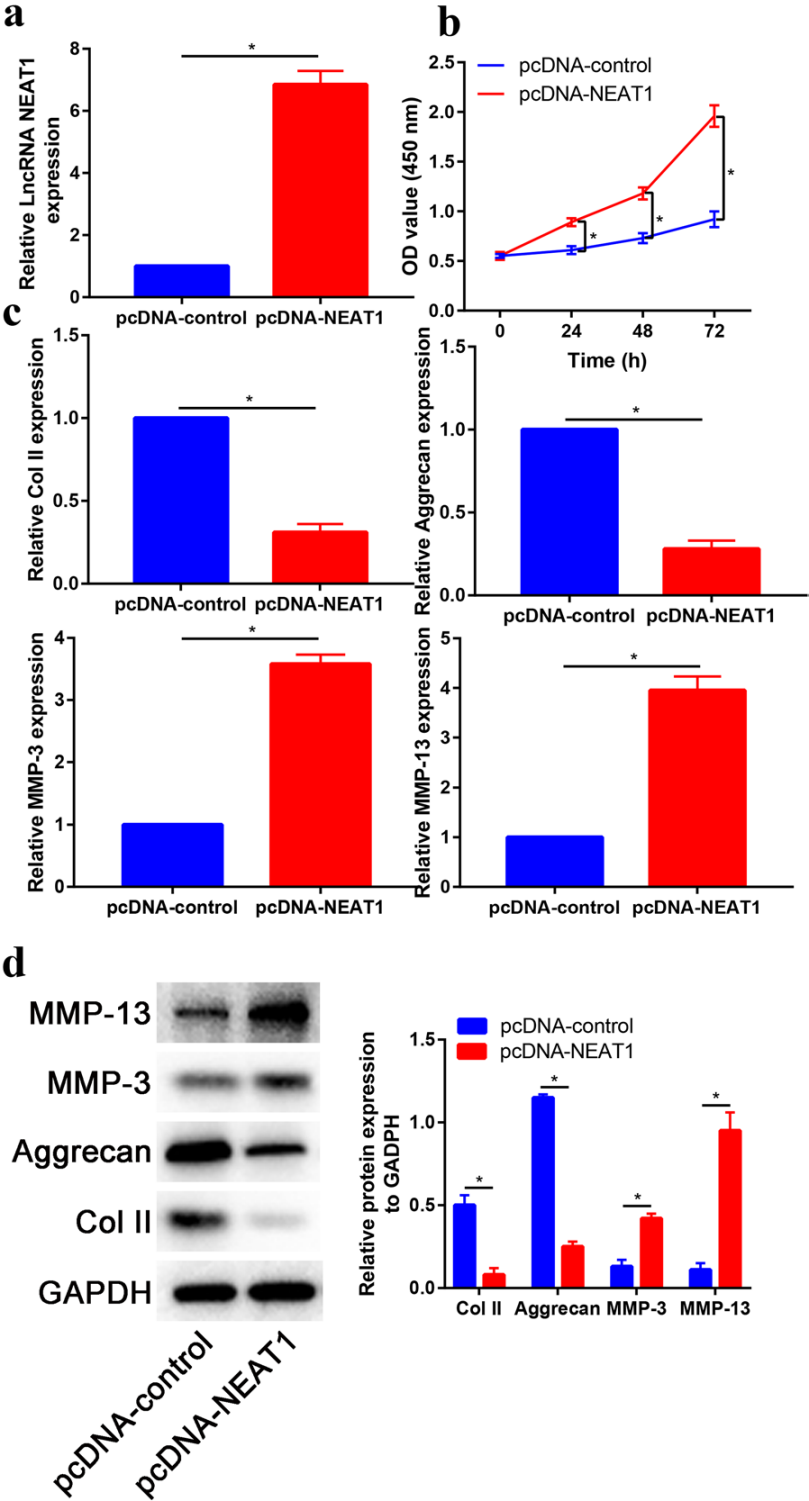
lncRNA NEAT1 induces the matrix degradation of nucleus pulposus cells. **a** Expression level of lncRNA NEAT1 in control and pcDNA-NEAT1 groups. **b** LncRNA NEAT1 promoted the proliferation of nucleus pulposus cells at 24, 48 and 72 h. **c** Expression of catabolic biomarkers (MMP-3 and MMP-13) and anabolic biomarkers (Col II and Aggrecan) in control and pcDNA-NEAT1 groups. **d** Western blot assay to measure the expression of catabolic biomarkers (MMP-3 and MMP-13) and anabolic biomarkers (Col II and Aggrecan) in control and pcDNA-NEAT1 groups. *p < 0.05

We then constructed a lncRNA NEAT1-overexpressing vector (pcDNA-NEAT1). Transfection efficiency were validated by quantitative real-time PCR (Fig. 3a). Cell proliferation was performed using CCK-8 assays at 24 h, 48 h and 72 h after transfection with pcDNA-NEAT1 (Fig. 3b). Overexpression of NEAT1 (pcDNA-NEAT1) enhanced cell proliferation at 24, 48 and 72 h post-transfection, as assessed by CCK-8 assay. Real-time PCR was performed to assess the catabolic biomarkers (MMP-3 and MMP-13) and anabolic biomarkers (Col II and Aggrecan) expression. Overexpression of lncRNA NEAT1 treatment promotes the mRNA expression of catabolic biomarkers (MMP-3 and MMP-13) and inhibits the mRNA expression of anabolic biomarkers (Col II and Aggrecan) expression (Fig. 3c). These results were further validated by Western blot assay (Fig. 3d).

### LncRNA NEAT1 inhibits Nrf2 to promote nucleus pulposus cell matrix degradation

**Fig. 4:**
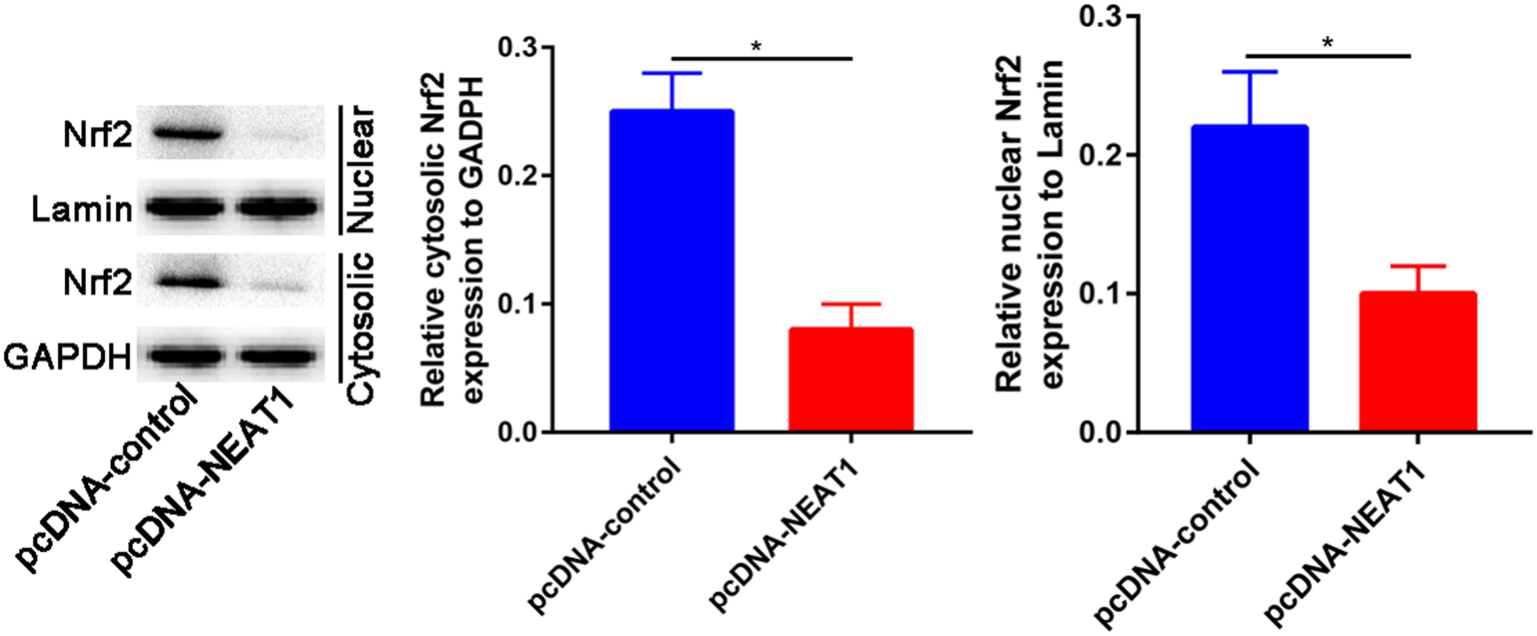
The expression of Nrf2 in the cytoplasm and nucleus of nucleus pulposus cells treated with control and pcDNA-NEAT1. *p < 0.05

The expression levels of Nrf2 in the nucleus and cytoplasm were assessed by Western blot assay. The Western blot results showed that overexpression of lncRNA NEAT1 inhibited the expression of Nrf2 in nucleus and cytoplasm in nucleus pulposus cells (Fig. 4).

**Fig. 5:**
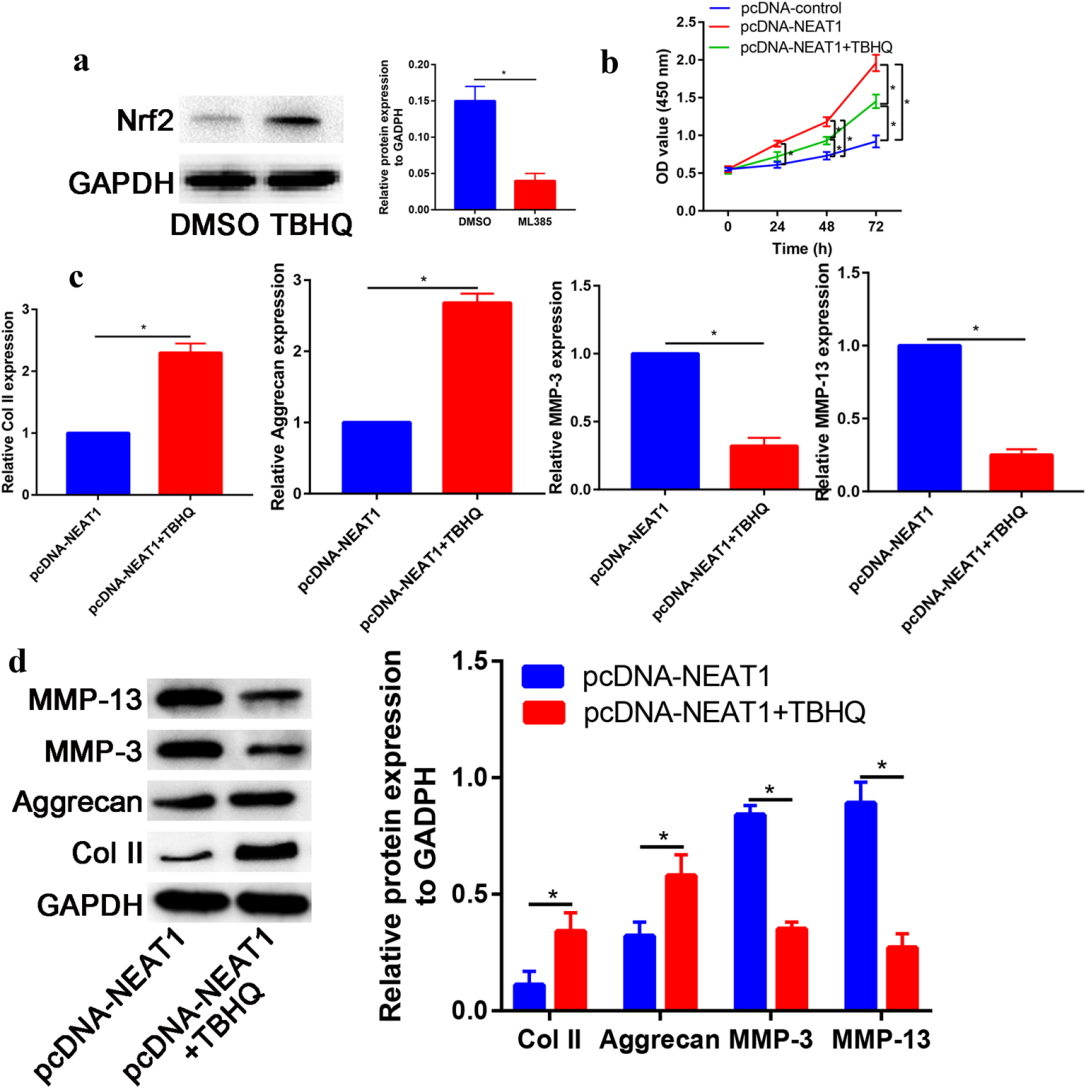
Nfr2 activator partially blocks the promotion effect of lncRNA NEAT1 in matrix degradation. **a** Nrf2 expression in control and Nfr2 activator (TBHQ) groups. **b** The effect of lncRNA NEAT1 with or without Nfr2 activator on nucleus pulposus cell proliferation evaluated by CCK-8 assay. **c** The effect of lncRNA NEAT1 with or without Nfr2 activator on mRNA expression of Col II, Aggrecan, MMP-3 and MMP-13 evaluated by RT-PCR. **d** The effect of lncRNA NEAT1 with or without Nfr2 activator on protein expression of Col II, Aggrecan, MMP-3 and MMP-13 evaluated by Western blot assay. *p < 0.05

To further evaluate the role of the Nrf2 signaling pathway in nucleus pulposus cell degradation, the Nrf2 signaling pathway activator TBHQ was used to investigate the effects of overexpression of lncRNA NEAT1 on IVD mediated by Nrf2 signaling. The Nrf2 expression increased significantly after pretreatment with Nrf2 activator (TBHQ, Fig. 5a). Nucleus pulposus cell proliferation induced by overexpression of lncRNA NEAT1 is partially blocked by TBHQ (Fig. 5b).

Compared with pcDNA-NEAT1, pretreatment with TBHQ following pcDNA-NEAT1 significantly increase anabolic biomarkers (Col II and Aggrecan) and decrease catabolic biomarkers (MMP-3 and MMP-13, Fig. 5c). These results were further validated by Western blot assay (Fig. 5d).

## Discussion

This study investigated the role and mechanism of lncRNA NEAT1 in occurrence and development of IVD. First, we searched GEO database and found that lncRNA NEAT1 was high expressed in the IVD tissue than normal tissue. LncRNA NEAT1 expression was up-regulated by IL-1β- and TNF-α-stimulated nucleus pulposus cells. Our results suggested that lncRNA NEAT1 expression was positively correlated with degree of degeneration grade. Overexpression of lncRNA NEAT1 significantly promotes the matrix degradation, which mainly through target with Nrf signaling pathway.

LncRNA NEAT1 plays an important role in many diseases, such as gastric cancer [24], prostate cancer [25], renal fibrosis [26], and osteosarcoma [27]. What is more, Liu et al. [28] found that knockdown of lncRNA NEAT1 inhibit SK-N-SH cells apoptosis, inflammation and cytotoxicity. We found that lncRNA NEAT1 was differentially expressed in IVD tissue and IL-1β/TNF-α stimulated nucleus pulposus cells. Thus, we speculated that lncRNA NEAT1 may affect the disc degeneration.

The functional roles of lncRNA NEAT1 in cells was analyzed by loss function assays. Overexpression of lncRNA NEAT1 significantly promotes the degradation of matrix. Moreover, lncRNA NEAT1 significantly down-regulated the Nrf2 expression in nucleus and cytoplasm. Those results suggested that lncRNA NEAT1 may target Nrf2 expression to regulate the matrix degradation.

Activation of the Nrf2 signaling pathway helps protect cells from oxidative stress [29, 30]. Oxidative stress could enhance the Nrf2 activity, and upon activation, it would translocate into the nucleus [18]. Tang et al. [31] found that a Nrf2 gene knockout leads to IVD degeneration, which mainly through regulation of autophagy in mouse model. Overexpression of lncRNA NEAT1 inactivated the Nrf/ARE signaling pathway. This result was corroborated by analyzing the expression of Nrf2 protein in the nucleus and cytoplasm. To explore this further, Nrf2 activator (TBHQ) was applied to determine whether lncRNA NEAT1 inactivated the Nrf2 signaling pathway to induce matrix degradation. Pretreatment with TBHQ following pcDNA-NEAT1 significantly increases anabolic biomarkers (Col II and Aggrecan) and decreases catabolic biomarkers (MMP-3 and MMP-13). These results suggested that lncRNA NEAT1 promotes matrix degradation through regulation Nrf/ARE signaling pathway.

## Conclusion

In short, we first elaborate the role and mechanism of lncRNA NEAT1 in IVD progression. We suggested that the lncRNA NEAT1 expression was up-regulated in degenerated NP tissues. Overexpression of lncRNA NEAT1 induced matrix degradation via modulating Nrf2/ARE signaling pathway. Our data proved that lncRNA NEAT1 may be useful as a new potential therapeutic target in IVD.

## Data Availability

We declare that the materials described in the manuscript will be freely available to all scientists for non-commercial purposes.

## Abbreviations

IVD: Intervertebral disc degeneration
GEO: Gene Expression Omnibus
TBHQ: Tert-Butylhydroquinone
LncRNA: Long intergenic noncoding RNA
NEAT1: Nuclear enriched abundant transcript 1
TNF-α: Tumor necrosis factor-alpha
IL-1β: Interleukin-1β
ANOVA: One-way analysis of variance
